# Identification of novel sesquiterpene synthase genes that mediate the biosynthesis of valerianol, which was an unknown ingredient of tea

**DOI:** 10.1038/s41598-018-30653-w

**Published:** 2018-08-20

**Authors:** Jun-ichiro Hattan, Kazutoshi Shindo, Tetsuya Sasaki, Fumina Ohno, Harukuni Tokuda, Kazuhiko Ishikawa, Norihiko Misawa

**Affiliations:** 1 Research Institute for Bioresources and Biotechnology, Ishikawa Prefectural University, 1-308 Suematsu, Nonoichi-shi, Ishikawa, 921-8836 Japan; 2 Department of Food and Nutrition, Japan Women’s University, 2-8-1 Mejirodai, Bunkyo-ku, Tokyo, 112-8681 Japan; 3 Industrial Research Institute of Ishikawa, 2-1 Kuratsuki, Kanazawa-shi, Ishikawa, 920-8203 Japan; 4 Department of Complementary and Alternative Medicine, Clinical R&D, Graduate School of Medical Science, Kanazawa University, 13-1 Takara-machi, Kanazawa-shi, Ishikawa, 920-8640 Japan; 5 National Institute of Advanced Industrial Science and Technology, 1-8-31 Midorigaoka, Ikeda-shi, Osaka 563-8577 Japan

## Abstract

Seven cDNA clones encoding terpene synthases (TPSs), their structures closely related to each other, were isolated from the flower of *Camellia hiemalis* (‘Kantsubaki’). Their putative TPS proteins were phylogenetically positioned in a sole clade with the TPSs of other *Camellia* species. The obtained *Tps* genes, one of which was designated *ChTps1* (*ChTps1a*), were introduced into mevalonate-pathway-engineered *Escherichia coli*, which carried the genes for utilizing acetoacetate as a substrate, and cultured in a medium including lithium acetoacetate. Volatile products generated in the *E*. *coli* cells transformed with *ChTps1* were purified from the cell suspension culture, and analyzed by NMR. Consequently, the predominant product with ChTPS1 was identified as valerianol, indicating that the *ChTps1* gene codes for valerianol synthase. This is the first report on a gene that can mediate the synthesis of valerianol. We next synthesized a *Tps* ortholog encoding ChTPS1variant R477H (named CsiTPS8), whose sequence had been isolated from a tea tree (*Camellia sinensis*), carried out similar culture experiment with the *E*. *coli* transformant including *CsiTps8*, and consequently found valerianol production equally. Furthermore, GC-MS analysis of several teas revealed that valerianol had been an unknown ingredient in green tea and black tea.

## Introduction

Floral scents consist of various volatile compounds of low molecular weight (30–300 Da) such as terpenes (monoterpenes and sesquiterpenes), aliphatics, benzenoids, and phenylpropanoids^1,2^. These compounds attract and guide pollinators^2–4^ and protect flowers against herbivores and pathogens^2,5^. Dobson reported terpenes provided an invitation for butterflies, moths, and bees in insect-pollinated flowers^6^. Terpenes, of which there are more than 55,000 known, are biosynthesized from C_5_ isopentenyl diphosphate (IPP) and its isomer dimethylallyl diphosphate (DMAPP)^7–9^. IPP and DMAPP are supplied by two independent pathways, the mevalonate (MVA) pathway and the methylerythritol phosphate (MEP) pathway. The former pathway exists in the cytosol of eukaryotes and in some actinobacteria and archaea, the latter in prokaryotes and the chloroplast of plants^10–12^. DMAPP is condensed with IPP to yield geranyl diphosphate (GPP; C_10_), which is then converted to farnesyl diphosphate (FPP; C_15_) with IPP, by prenyltransferases. Terpene synthases (TPSs; also called terpene cyclases) release diphosphate from GPP and FPP, and the remaining reactive carbocation intermediates are intra-molecularly cyclized in almost all cases, yielding monoterpenes and sesquiterpenes, respectively^13^. When TPSs initiate these reactions, cleaving diphosphate, aspartic acid (D)-rich motifs of TPS interact with diphosphate through intervening three Mg^2+^ ions and water molecules^14–16^. Therefore, D-rich motifs, such as the RDR, DDxxD, and NSE/DTE motifs, are indispensable for TPS function. *Tps* genes, especially higher plant-derived ones, have been characterized extensively^14,17^, while cytochromes P450 have been shown to metabolize volatile terpenes generated with TPSs^17^.

Camellia plants are flowering trees beloved all over the world. A large variety of cultivars have been bred for their flowers, and a few breeding has been carried out on their flower fragrances. Camellia trees grow spontaneously in Japan and other East Asian countries^18,19^. *Camellia japonica*, called ‘Tsubaki’ in Japan, is found all over Japan except for Hokkaido, whereas *C*. *sasanqua*, called ‘Sazanka’, is dominantly present from Okinawa to Yamaguchi Prefectures^20^. The breeding of *C*. *japonica* as an ornamental has long been performed^21^. Most flowers of *C*. *japonica* are poorly fragrant^18^. *C*. *japonica* has also been crossed with *C*. *lutchuensis*, which emits the strongest fragrance in the genus *Camellia*, to produce fragrant Camellia plants^22^. The crossbred lines of *C*. *sasanqua* have also been produced^20^. *C*. *hiemalis*, called ‘Kantsubaki’, is considered to have originated as an interspecific hybrid between *C*. *japonica* and *C*. *sasanqua*, by isozyme analysis^23^ and morphological and cytological studies^24–26^. A comparison of the essential oil amounts and compositions showed that *C*. *hiemalis* and *C*. *sasanqua* were quite similar species^27^. The floral scents of *C*. *japonica*, *C*. *sasanqua*, *C*. *hiemalis*, and other *Camellia* species have been analyzed by gas chromatography-mass spectrometry (GC-MS)^18,28^, indicating the presence of various monoterpenes and sesquiterpenes. Green tea that is produced of leaf buds of *Camellia sinensis* was also analyzed by GC-MS, and was found to contain terpenes along with some unknown compounds^29^.

We have identified new terpene synthase (*Tps*) genes that can mediate the synthesis of volatile sesquiterpenes or monoterpenes in several fragrant plants, which belong to the families Araliaceae, Zingiberaceae, and Camelliaceae^30–34^. As for the family Camelliaceae, we identified the hedycaryol synthase gene (*CbTps1*) in *Camellia brevistyla* and the linalool synthase (*CsTps1*) gene in *Camellia saluenensis*^30^. Here, we report GC-MS analysis of the floral scent of *C*. *hiemalis*, isolation and functional analysis of *Tps* genes from the flower (Supplementary Fig. S1), with our original functional analysis system using an MVA-pathway-engineered *E*. *coli* strain that can utilize acetoacetate as a substrate^35^. Furthermore, we report the function of a *Tps* gene derived from the leaf buds of a tea tree *C*. *sinensis*.

## Results

### GC-MS analysis of *Camellia hiemalis* floral scent

About 30 years have passed since *C*. *hiemalis* volatile compounds were analyzed by Omata *et al*.^28^. Therefore, we performed a qualitative analysis of the floral scent of *C*. *hiemalis* by GC-MS (Supplementary Table S1). The most abundant component was a phenyl propanoid, eugenol, which comprised more than 80% of the total floral scent compounds. Monoterpenes and sesquiterpenes were also detected, and these volatile terpenes accounted for about 10%, largely consisting of linalool and its derivatives. This result agreed with that of Omata *et al*.^28^, which detected linalool and linalool oxide as the major compounds in *C*. *hiemalis* cultivars. Sesquiterpene elemol and guaiol were detected as the minor components.

### Isolation of *ChTps1* and its paralogs from Camellia flower

Since the existence of several volatile terpenes in the floral scent was reconfirmed by the GC-MS analysis, we decided to isolate *Tps* (*ChTps*) genes from *C*. *hiemalis*. For their isolation, degenerate primers designed based on the nucleotide sequences deduced from DDxxD motif (described above) were used. A partial *Tps* cDNA fragment including its 5′-end was obtained by degenerate RT-PCR, using cDNA prepared from a *C*. *hiemalis* flower as the template. Subsequently, a full-length *Tps* sequence was acquired by performing 3′-RACE. Depending on this sequence, 5′ and 3′-end primers were synthesized, and more than 20 full-length *Tps* clones were isolated by PCR with high fidelity. These sequences were analyzed based on multiplicity of clones with the same sequence or the formation of an ORF, classified into seven genes, and designated *ChTps1a*, *ChTps1b*, *ChTps1c*, *ChTps1d*, *ChTps1e*, *ChTps1f*, and *ChTps1g*, which have several substitutions [or a deletion (*ChTps1d*)] (Table 1). *ChTps1a* was also named *ChTps1*, since it was found to be the representative corresponding gene as analyzed later. Four, three, and four clones had the same sequence to those of *ChTps1a*, *ChTps1b*, and *ChTps1c*, respectively. As for *ChTps1d*, *ChTps1e*, *ChTps1f*, and *ChTps1g*, *o*nly one clone each was obtained. These genes were inserted into the expression vector, introduced into *E*. *coli* together with plasmid pAC-Mev/Scidi/Aacl^36^, and the amount of TPS products was compared (mentioned below). The clone *ChTps1a* showed the highest enzyme activity. Therefore, we designated this clone as *ChTps1* and the encoded protein as ChTPS1. Supplemental Fig. S2 shows the alignment of ChTPS1 and other TPS amino acid sequences isolated by our group. ChTPS1 consisted of 554 amino acids (aa) with a calculated molecular mass of 64.1 kDa. D-rich motifs, such as the RDR (270–272), DDxxD (307–311), and NSE/DTE (452–460) motifs, are conserved. No transit peptide was found at the N-terminus of the ChTPS1 protein by the iPSORT^37^ prediction.

**Table 1:** Amino acid substitutions and a deletion observed in the 7 ChTPS1 clones.

clone	amino acid	42	68	78	139	145	185	290	295	317	319	321	365	395	396–430	399	443	477	503	541
ChTPS1a		W	A	E	G	V	G	S	V	D	L	L	**D**	A		E	A	**R**	M	K
ChTPS1e		W	A	E	G	**M**	G	**F**	V	D	L	L	**D**	A		**A**	A	H	**I**	K
ChTPS1c		W	A	E	**C**	V	**E**	S	**G**	**N**	L	L	A	A		E	A	H	M	K
ChTPS1f		W	A	E	**C**	V	**E**	S	**G**	**N**	L	L	A	A		E	A	H	M	**N**
ChTPS1b		W	A	**Q**	G	**M**	**E**	S	V	**N**	L	**I**	A	A		E	A	H	M	K
ChTPS1g		**S**	**V**	E	G	V	G	S	V	D	**V**	L	A	**V**		E	**T**	H	M	K
ChTPS1d		**S**	**V**	E	G	V	G	S	V	D	**V**	L	A	**V**	deletion	—	**T**	H	M	K

Substituted (or minor) amino acids are highlighted in bold. Amino acids from 396 to 430 were deleted in clone d. The order of the clones was rearranged depending on their substitution similarity.

### Phylogenetic analysis of *ChTPS1*

**Figure 1:**
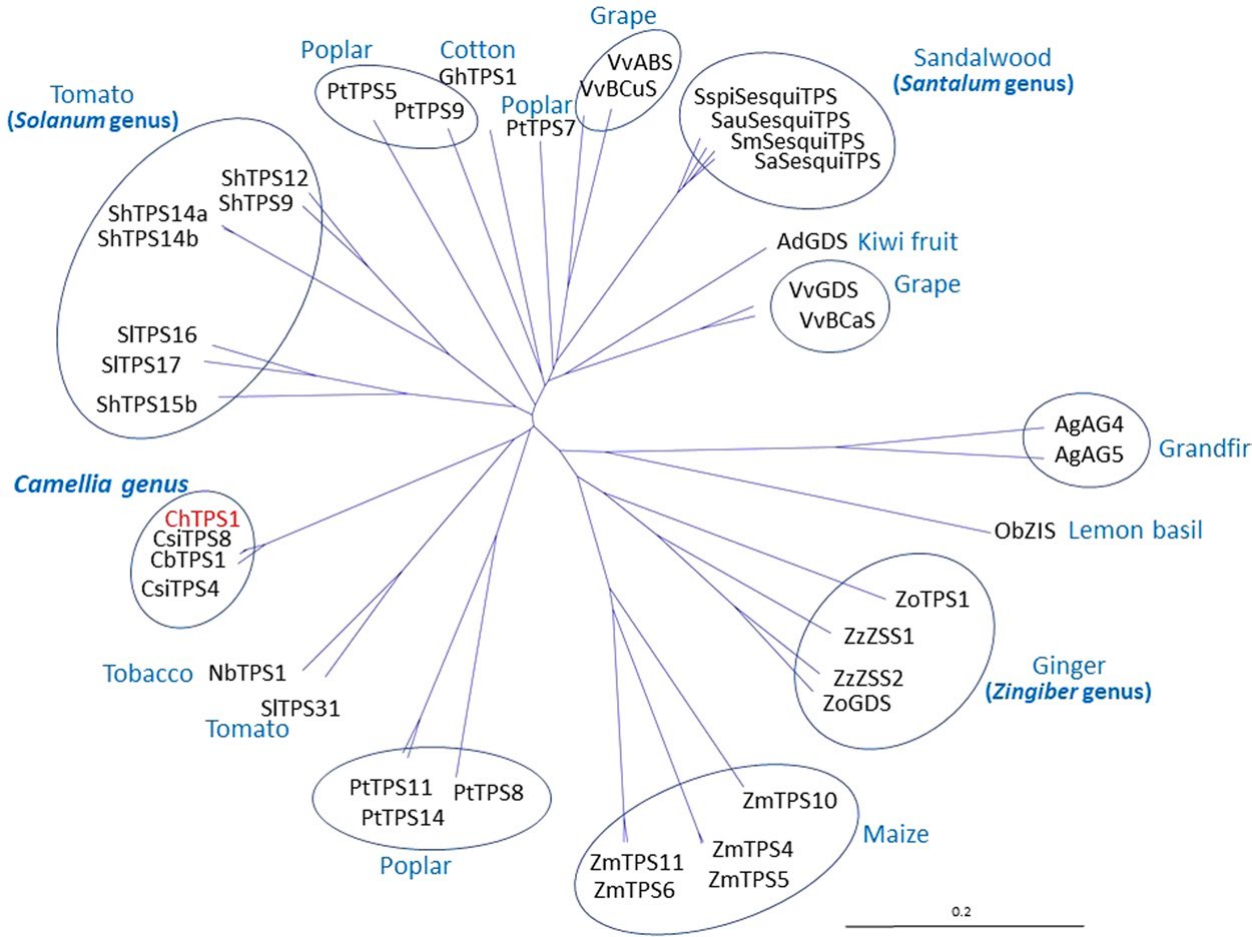
Phylogenetic tree of plant sesquiterpene synthases. ChTPS1 and known sesquiterpene synthases (TPSs) were analyzed phylogenetically. TPS accession numbers are as follows: VvABS (NP_001267972), VvBCaS (ADR74193), VvBCuS (ADR74200), VvGDS (Q6Q3H3), AdGDS (AAX16121), ZzZSS1 (BAG12020), ZzZSS2 (BAG12021), ZoGDS (AAX40665), ZoTPS1 (BAI67934), ZmTPS4 (AAS88571), ZmTPS5 (AAS88574), ZmTPS6 (AAS88576), ZmTPS10 (AAX99146), ZmTPS11 (ACF58240), ObZIS (AAV63788), AgAG4 (AAC05727), AgAG5 (AAC05728), PtTPS5 (KF776503), PtTPS7 (KF776505), PtTPS8 (KF776506), PtTPS9 (KF776507), PtTPS11 (KF776509), PtTPS14 (KF776512), SaSesquiTPS (ACF24768), SauSesquiTPS (HQ343281), SmSesquiTPS (JF746810), SspiSesquiTPS (HQ343282), NbTPS1 (AHM10157), GhTPS1 (AFQ23183), ShTPS9 (AEM23825), ShTPS12 (AEM23826), ShTPS14a (AEM23827), ShTPS14b (AEM23829), ShTPS15b (AEM23830), SlTPS16 (AEM23831), SlTPS17 (AEM23832), SlTPS31 (AEM23833), CsiTPS4 (ANB66343), CsiTPS8 (ANB66333), CbTPS1 (BAU68096). Circles indicate TPSs in the same genera or the same species.

The ChTPS1 protein was analyzed phylogenetically by comparison with known plant TPSs registered in the NCBI database (Fig. 1). TPS proteins derived from the same plant species or family tended to fall into the same clade, except for Poplar TPSs. ChTPS1 was phylogenetically positioned with the TPSs of other *Camellia* species in a sole-gene clade. The amino acid sequence of *Camellia sinensis* TPS8 (CsiTPS8) was quite similar to that of ChTPS1 and had only one amino acid substitution (R477H) except for the N-terminal extension. CbTPS1, which we have isolated and reported as hedycaryol synthase recently^30^, was also similar to ChTPS1, and 9 amino acid substitutions existed (Supplemental Fig. S2).

### Functional characterization of *ChTPS1* and its paralogs in the mevalonate-pathway-engineered *E. coli*

**Figure 2:**
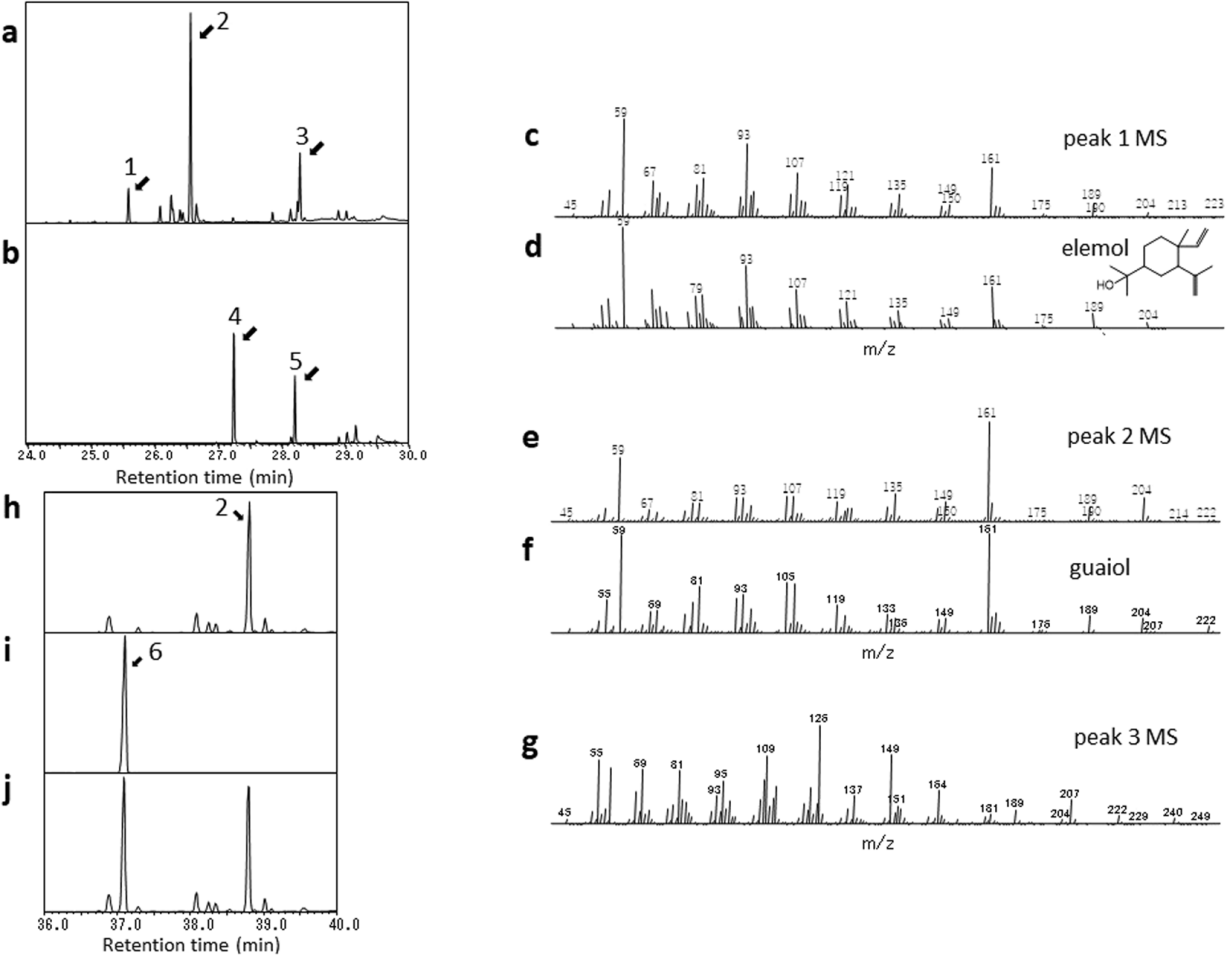
Gas chromatography-mass spectrometry (GC-MS) analysis of the ChTPS1 product. The ChTPS1 product extracted from *E*. *coli* (pRSF-ChTps1a and pAC-Mev/Scidi/Aacl) was subjected to GC-MS. Total ion chromatograms (TIC) of the ChTPS1 product (**a**) and of the empty vector product (control) (**b**) MS of Peak 1 (**c**) MS of elemol in the database (**d**) MS of Peak 2 (**e**) MS of guaiol in the database (**f**) MS of Peak 3 (**g**) TIC around the ChTPS1 Peak 2 area (**h**) TIC of the authentic guaiol standard (**i**) and TIC of the ChTPS1 product and guaiol mixture (**j**). As for the GC-MS analysis conditions, such as the heating-up program, for (**h**,**i** and **j**) see ref.^36^.

**Figure 3:**
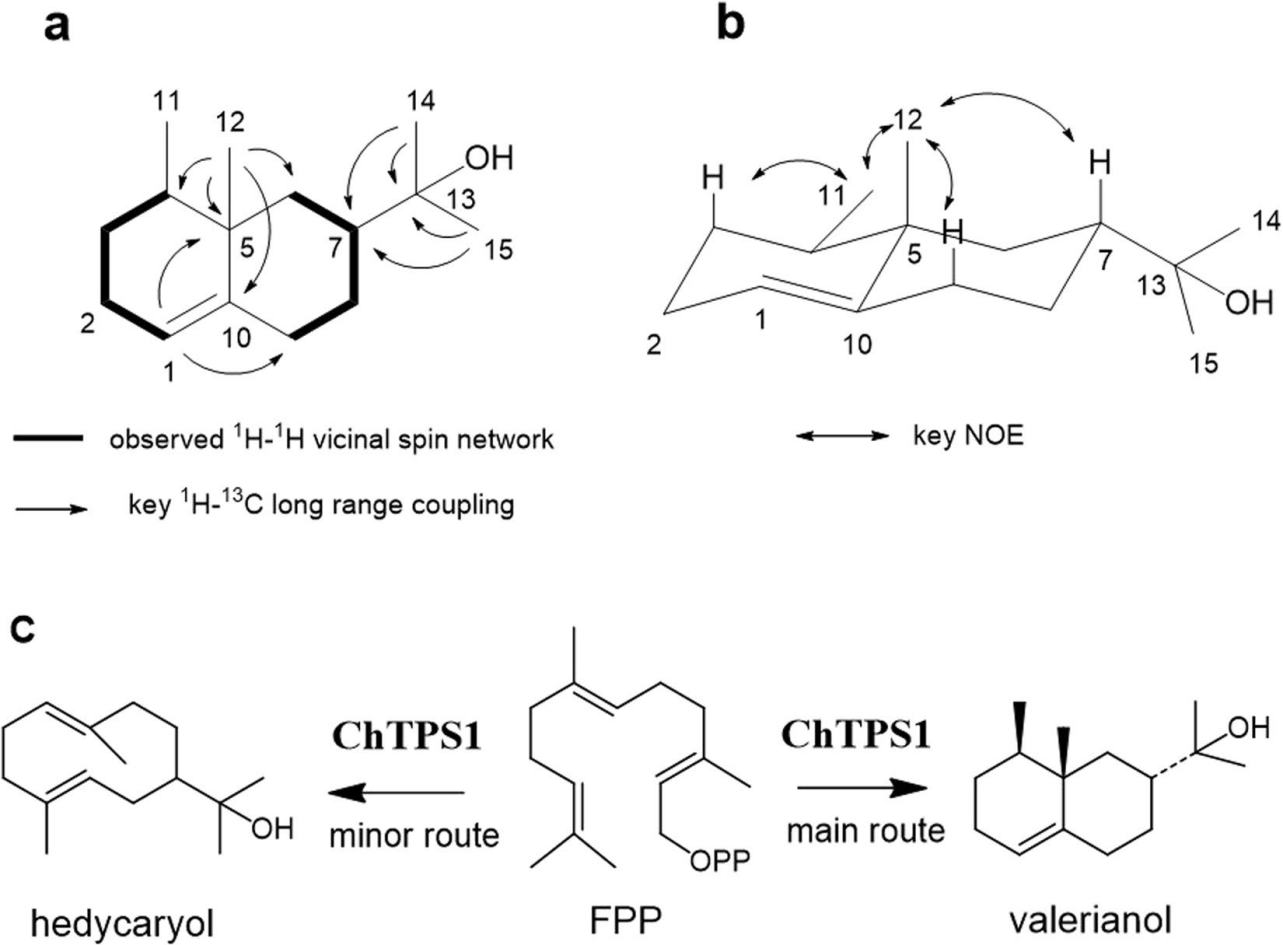
^1^H and ^13^C NMR and HSQC spectral analysis of the Peak 2 compound (**a**) its relative configuration by NOEs (**b**) and the catalytic function of ChTPS1 (**c**).

As mentioned above, seven *ChTps1a-g* clones were individually expressed in the MVA-pathway-engineered *E*. *coli* that can utilize acetoacetate as a substrate^35^, and sesquiterpenes synthesized by each clone were analyzed by GC-MS. An empty vector clone showed two peaks (Fig. 2b). The predicted compounds of Peaks 4 and 5 were farnesol and farnesyl acetate, respectively, which are likely to be derived from FPP, and were found to disappear in the ChTps1 products, generating several new peaks, instead (Fig. 2a). Thus, we focused on three new distinct peaks (Peaks 1, 2, and 3) that were considered to be the ChTPS1 (ChTPS1a) products. The MS of the predominant product (Peak 2) had the highest similarity to that of guaiol in the MS database (Fig. 2e,f). We then mixed authentic guaiol with the ChTPS1 products, and carried out GC-MS analysis. As a result, the peaks of Peak 2 and guaiol were not overlapped, indicating that Peak 2 was not guaiol (Fig. 2h–j). Thus, the Peak 2 compound was purified, and subjected to 1D (^1^H and ^13^C) and 2D (^1^H-^1^H COSY, HSQC, HMBC, and NOESY) NMR spectroscopic analyses (in CDCl_3_). ^1^H and ^13^C NMR and HSQC spectral analysis of the Peak 2 compound confirmed that it possessed 4 methyls, 5 sp^3^ methylenes, 2 sp^3^ methines, 2 sp^3^ quarternary carbons, 1 sp^2^ methine, and 1 sp^2^ quarternary carbon. Further, two vicinal ^1^H spin networks of H-1-H-11 and H-6 to H-9 (bold line in Fig. 3a) observed in the ^1^H-^1^H COSY spectrum and the key ^1^H-^13^C long range couplings observed in the HMBC spectrum (arrows in Fig. 3a) proved the planar structure of the Peak 2 compound as shown in Fig. 3a. The relative configuration of the Peak 2 compound was analyzed by NOESY spectrum, and the observed key NOEs (arrows in Fig. 3b) proved the relative configuration of the Peak 2 compound as shown in Fig. 3b. From these observations, the Peak 2 compound was identified to be valerianol. These results indicated that ChTPS1 synthesizes valerianol as the predominant product. The MS of Peak 1 (minor) coincided to that of elemol, while the MS of Peak 3 (minor) was an unknown sesquiterpene, (Fig. 2c,d,g). In order to check whether Peak 1 was elemol or not, we mixed authentic elemol with the ChTPS1 products, and carried out GC-MS analysis. Consequently, the peaks of Peak 1 and elemol were overlapped, indicating that the Peak 1 GC product was elemol (data not shown). Elemol was reported to be a thermal rearrangement product from hedycaryol^30,38^. It is therefore likely that elemol originated from hedycaryol during the heating-up process of GC-MS. The Peak 1 compound was confirmed to be hedycaryol by HPLC analysis, the same as described previously (data not shown)^30^. The plausible conformations of hedycaryol were reported by Faraldos *et al*.^39^ Based on these results, *ChTps1* (*ChTps1a*) was identified as a novel gene encoding valerianol [valerianol/hedycaryol (minor)] synthase (Fig. 3c). This is the first report on a gene that can mediate the synthesis of valerianol.

**Figure 4:**
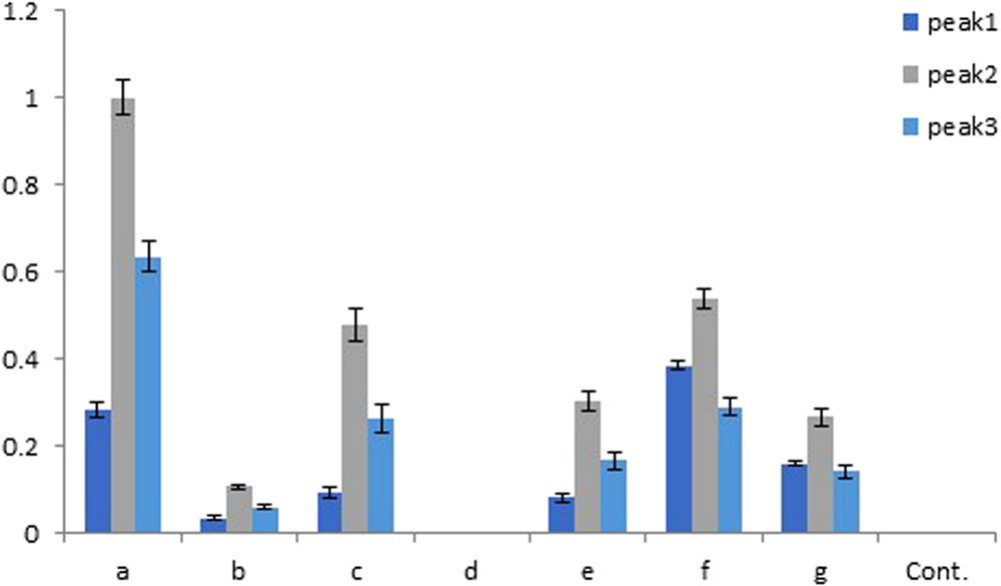
Comparison of the peak areas obtained by GC-MS analysis of the product of 7 *ChTps1* clones. Three peak areas, shown in Fig. 2a (Peaks 1–3), of total ion chromatogram were obtained by GC-MS analysis of the ChTPS1 product. These peak areas of 7 *ChTps1* clones (**a**–**g**, shown in Table 1) individually were compared. Relative abundance of each peak was depicted when the Peak 2 area of *ChTps1a* clone was set to 1. Bars represent means ± standard deviation of three replicates. Cont. stands for empty vector control.

The product amounts of all ChTPS1 substitution clones were less than ChTPS1 (ChTPS1a) (Fig. 4). In comparison with peak areas of each clone (Fig. 4), Peaks 1 and 3 were the third and second largest abundant products, respectively, in ChTPS1a, b, c, and e. As for the ChTPS1f product, the second and third most abundant products were in reverse order. These two sesquiterpenes were contained equally in the product of ChTPS1g. As for ChTPS1d, no TPS product was observed. These differences in each ChTPS1 product amount were attributed to the differences in amino acid sequences (Table 1). For example, Peak 2 valerianol amounts of ChTPS1c and f were almost the same, but the Peak1 hedycaryol amount of ChTPS1f was about 4 times higher than that of ChTPS1c (Fig. 4). This difference in the ChTPS1f enzyme activity was induced by the single amino acid substitution K541N (Table 1). ChTPS1g and d have a similar amino acid sequence except for a deletion from the 396 to 430 amino acid region of ChTPS1d (Table 1), which was the cause of inactivation (Fig. 4).

### Structure-activity relationship studies of *ChTPS1*

The plausible 3D structural model of ChTPS1 was built by SWISS-MODEL^40^ using the pdb of Tobacco 5-epi-aristolochene synthase^16^ as the template. Then the ChTPS1 3D model was applied to predict the effect of amino acid substitutions (Supplementary Table S2). The substitution sites were roughly divided into 3 parts: surface, internal and loop. Substitutions on the molecular surface would have a little effect on the enzyme activity, whereas those exist in the internal part might have some effect interacting with neighboring residues. The loop, which means β-turn between α-helices, sometimes locates at the surface of the molecule and contributes to determine the molecular structure. Hence, substitutions in the loop would change the structure and have severe effects in some cases. Supplementary Table S2 summarized these effects of substitutions in the ChTPS1 clones. Intriguingly, ChTPS1 clones contained 4 substitutions in the loops and 2 of them showed characteristic changes of the enzyme activity. One was K541N, which increased the Peak 1 ratio, mentioned above, and the other was R477H (see below). R477 is located at the loop and forms an ionic bond with E487 located at the α-helices based on the model structure. And the α-helices (483–493) is forming the hydrophobic interaction with the next α-helices (440–461) containing one of the the TPS-conserved motifs (Supplementary Fig. S2).

### Expression analysis of *ChTps1*

Tissue specific *ChTps1* expression was analyzed by RT-PCR using total RNAs extracted from the flowers, leaves, and stems. After the amplification of 35 PCR cycles, the concentration of the amplified *ChTps1* fragment was compared visually (Supplementary Fig. S3). The amount of *ChTps1* transcript was high in the flowers, but quite low in the leaves and stems. Thus, *ChTps1* expression was flower specific.

### Functional characterization of CsiTPS8

As mentioned above, we found that the amino acid sequence of *Camellia sinensis* CsiTPS8 was quite similar to that of ChTPS1 (ChTPS1a) (Fig. 1). CsiTPS8 had only one amino acid substitution R477H, which located in the loop structure (Supplementary Fig. S4). Since the product of CsiTPS8 was not reported in the NCBI database, we prepared a substituted construct encoding ChTPS1 variant R477H, which has the same amino acid sequence with that of CsiTPS8, and analyzed the product extracted from the transformed *E*. *coli*. We consequently found that the products of ChTPS1 (R477H) were the same as that of ChTPS1, and the primary product was valerianol (Supplementary Fig. S5). The product amounts of the R477H clone averaged 40 percent increase in comparison with that of ChTPS1 (Supplementary Fig. S6). As for valerianol (the peak 2 area), 46.5 ± 6.0% (n = 3) increased.

### GC-MS analysis of volatile compounds in green tea, roasted stem tea, and black tea

**Figure 5:**
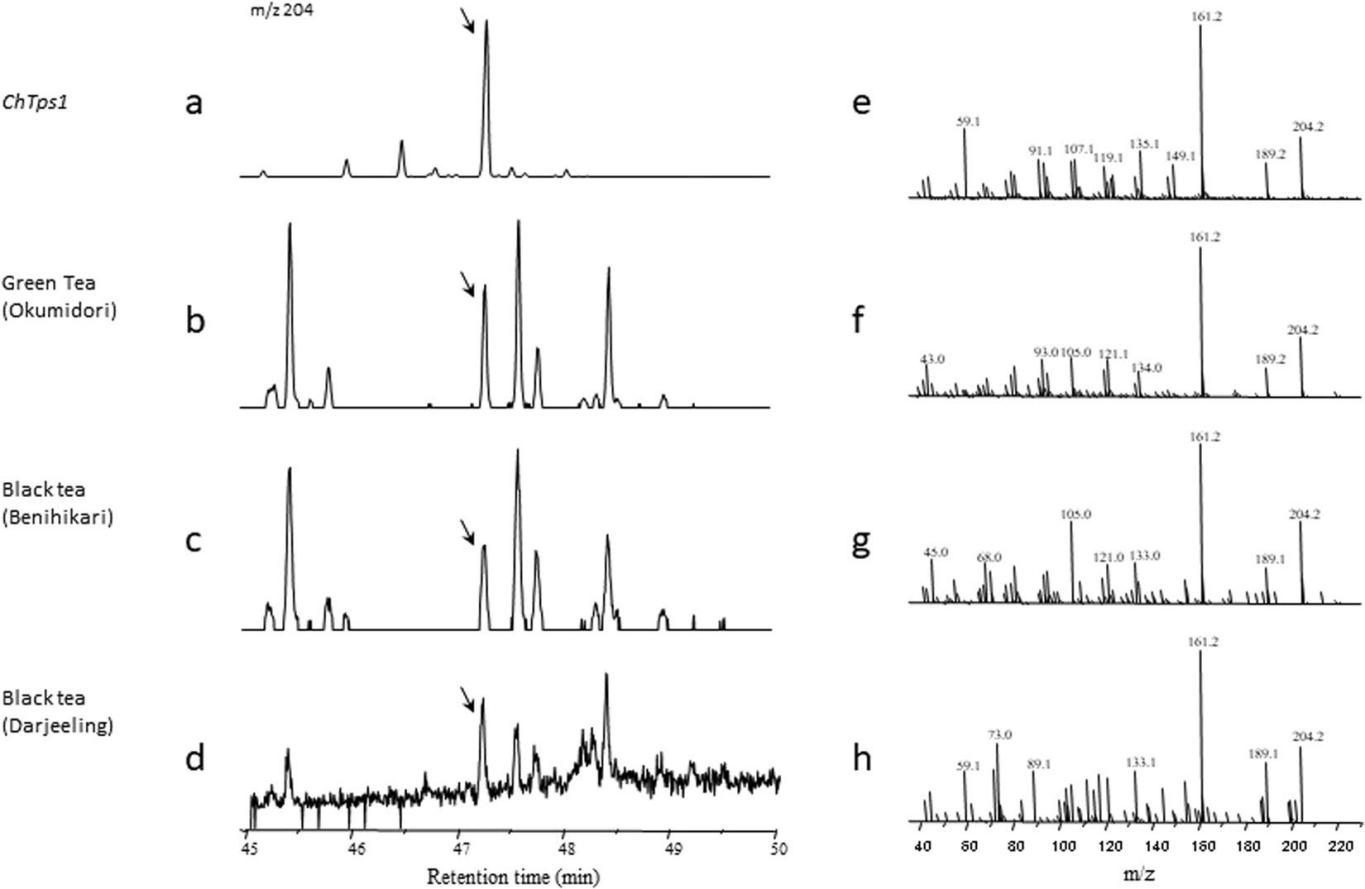
GC-MS analysis of the volatile compounds extracted from teas. Extracted ion chromatogram of m/z 204 (left) and MS of peaks detected at 47.3 min (right) were obtained by GC-MS analysis of green tea and black tea. Extracted samples were as follows: *ChTps1* clone (**a** and **e**) green tea (Okumidori) (**b** and **f**) Japanese black tea (Benihikari) (**c** and **g**) Indian black tea (Darjeeling) (**d** and **h**). Arrows indicate the peak at 47.3 min, where ChTPS1 main product valerianol was detected in (**a**).

Various teas, such as green tea and black tea, are consumed widely^41^. Many researchers have analyzed fragrances of these teas, which contain several unknown compounds^29,41^. Since tea is extracted from young leaves (and stems) of *C*. *sinensis*, which has CsiTPS8, we expected that one of the unknown compounds in tea is valerianol. Figure 5 shows the result of GC-MS analyses of these teas. Valerianol was detected in green tea and black tea, although the peak intensities of valerianol in both teas were about 1/1000 of that in the recombinant *E*. *coli* extract (data not shown).

### Inhibitory effects of sesquiterpenes on induction of EBV-EA activation by TPA

We finally carried out *in vitro* functional analysis of the purified valerianol against tumor promoter activity, since there were no reports on its physiological functions. As such a screening study for anti-tumor promoters, valerianol and other several sesquiterpenes, β-eudesmol, elemol, α-selinene, and γ-amorphene, were examined for their inhibitory effects on induction of Epstein-Barr virus-early antigen (EBV-EA) activation in Raji cells by 12-*O*-tetradecanoylphorbol-13-acetate (TPA)^42^. As a result, all the sesquiterpenes exhibited moderate inhibitory activity with very weak cytotoxicity on Raji cells, as shown in Table 2. Valerianol showed the strongest inhibitory activity among the examined sesquiterpenes.

**Table 2:** Inhibitory effects of treatment with sesquiterpenes on induction of EBV-EA activation by TPA

Sample^c^	Concentration^a^				
	1000	500	100	10	IC_50_
β-eudesmol	3.6 (60) ± 0.5^b^	50.4 ± 1.6	71.1 ± 2.2	97.0 ± 0.5	311 μM
elemol	5.8 (70) ± 0.4	52.6 ± 1.3	73.0 ± 2.4	100 ± 0.5	372
valerianol	2.1 (60) ± 0.4	46.5 ± 1.5	70.0 ± 2.5	95.6 ± 0.6	300
α-selinene	7.3 (60) ± 0.6	54.2 ± 1.5	74.4 ± 2.7	10 ± 0.3	382
γ-amorphene	8.1 (60) ± 0.6	55.6 ± 1.7	76.1 ± 2.5	100 ± 0.4	395

^a^Mol ratio to TPA (32 pmol/ml) = 100%. ^b^Values represent percentages relative to the positive control value (100%). Values in parentheses are the viability percentages of Raji cells. ^c^β-eudesmol,α-selinene, and γ-amorphene were purified from the cultures of recombinant *E. coli* strain that possessed the *Tps* genes mediating the production of respective sesquiterpenes^58^. Elemol was a gift from Takasago International Corporation (Tokyo, Japan).

## Discussion

*C*. *japonica* usually emits little floral scent^18^, while *C*. *sasanqua* and *C*. *hiemalis* smell fresh^28^. Several morphological and biochemical studies suggested *C*. *hiemalis* would be generated by crossbreeding of *C*. *sasanqua* and *C*. *japonica*^23^. These studies indicate scent-producing genes in *C*. *hiemalis* are derived from *C*. *sasanqua*. We have recently isolated the terpene synthase gene *CbTps1*^30^ from wild type *C*. *brevistyla*, the sequence of which was highly similar to that of *C*. *hiemalis ChTps1*. The floral scent compound hedycaryol was synthesized by CbTPS1 and ChTPS1. *C*. *brevistyla* and *C*. *sasanqua* had some common morphological aspects, such as flowers with a wide gap between heart-shaped petals^20^. Therefore, *C*. *sasanqua* could be a close relative of *C*. *brevistyla*. On the other hand, *C*. *sinensis* had *ChTps1*-like *CsiTps8* (Fig. 1), the DNA sequence of which included only 3 single nucleotide substitutions, one of which caused an amino acid substitution. Although Wachira *et al*.^43^ once reported that *C*. *sinensis* was phylogenetically distant from *C*. *sasanqua* and *C*. *japonica*, Caser *et al*.^44^ showed afterward that *C*. *sinensis* was a close relative of *C*. *sasanqua*. The latter data let us assume that these genes were shared from the common ancestor of *C*. *sinensis* and *C*. *sasanqua*. As for the natural habitat, *C*. *sinensis* originated in Southwestern China, Yunnan Province^45^, whereas *C*. *brevistyla* in Southeast China^46^. *C*. *sasanqua* is present from Okinawa to Kyusyu and Yamaguchi Prefecture, the southern part of Japan^20^.Taken together, the common ancestral *Camellia* of *C*. *sinensis*, *C*. *brevistyla*, and *C*. *sasanqua* could have existed in the southern part of China and possessed a *ChTps1*-like gene.

GC-MS analysis of the ChTPS1 product showed many peaks (including smaller ones around Peak 2) which were not observed in the control product (Fig. 2a,b). These peaks were expected to be sesquiterpenes, since farnesol and farnesyl acetate (Peaks 4 and 5) derived from FPP had disappeared. Actually, Peaks 1 and 2 were sesquiterpene hedycaryol and valerianol, respectively, and these sesquiterpenes were detected as elemol and guaiol in the floral volatiles (Supplementary Table S1). On the other hand, we were not able to determine the structure of Peak 3. The MS of Peak 3 showed the existence of fragments 204, 222, and 240, which might mean the binding of two water molecules. The NMR analysis revealed that the ChTPS1main product observed in the chromatogram of GC-MS analysis was valerianol. Hence, *ChTps1* was defined as the valerianol synthase gene.

In this study, we think that *ChTps1* and its six paralogs were isolated. However, this result may raise the following questions: Is such a multiple gene redundancy present in the higher plant, or are there results of the PCR error? Indeed, the redundancy of *Tps* genes has been observed in other plants, e.g., the grapevine genome analysis showed that there are 69 putatively functional *VvTps*, 20 partial *VvTps*, 63 *VvTps* probable pseudogenes, total 152 *VvTps* loci, of which 129 (85%) loci are distributed throughout 13 clusters covering 2 to 45 loci^14^. To check whether these loci contain homologous *VvTps*, whose amino acid sequences are almost identical, we tried BLAST search using VvTPS57 (Accession No. HM807394)^14^ as an example. As a result, VvTPS57 was 99–98% identical with about ten VvTPSs (data not shown). Figure 1 showed that Maize also contains multiple ZmTPSs. We similarly tried BLAST search using ZmTPS4, and it was found 99–98% identical with five ZmTPSs (data not shown). The reason why the grapevine genome contains many *VvTps*s is due to the clusters of closely related genes that can evolve as the result of unequal cross-over^14^. Moreover, we can point out that the increased ploidy would be one of the causes of the redundancy. Grapevine is described as a ‘palaeo-hexaploid’, which has a diploid content that corresponds to the three full diploid contents of the three ancestors^47^. Whereas maize is tetraploid^48^ and *C*. *hiemalis* is hexaploid (2n = 6x = 90)^49^. If unequal cross-over and duplication occurs in such plants and some trait is selected in breeding, the number of relating genes will increase. In fact, the relative abundance of terpenoids in grapevine is directly correlated with the aromatic features of wines^50^. Supplemental Table S1 suggested the existence of additional ChTPSs, such as linalool synthase, germacrene A (transformed into β-elemene by heating) synthase, and β-eudesmol synthase. The locus number of *ChTps*s might have been increased through the breeding selection based on the floral fragrance.

In the cloning process, we used DNA polymerase PrimeSTAR GXL, which has higher fidelity and causes one mismatched base per about 16,200 bases. Since *ChTps1* is composed of 1,665 bases, about 0.1 mismatched base is expected to generate per one clone. Moreover, we adapted the PCR steps with 25 cycles to decrease the mismatch, while the manufacturer’s instruction recommended the PCR steps with 30 cycles. Table 1 showed that ChTPS1s had 6–7 amino acid substitutions comparing with ChTPS1a, and nucleotide sequences of *ChTps1s* included 8–12 base substitutions (data not shown). If all these nucleotide substitutions were induced by the PCR error, the polymerase must have caused errors at least 100 times higher frequency. It is difficult to increase the mismatch rate without increasing the concentration of the magnesium ion and the manganese ion and/or changing the contents ratio of dNTP^51^. Supplemental Fig. S7 shows the phylogenetic tree of the nucleotide sequences of seven *ChTps1* genes. *ChTps1f* was phylogenetically close to *ChTps1c*, and these clones have one base pair difference. This may be caused by the PCR error. However, the other *ChTps1s* were mutually more distant, and the nucleotide substitutions were accumulated in each clone. Moreover, as for *ChTps1a-c*, the same multiple clones were isolated. Taken together, these results suggest that the *ChTps1* variation (except *ChTps1f*) was not induced by the PCR error but reflecting the aspect of the *C*. *hiemalis* genome that has repeated duplication and mutation.

We analyzed TPS activities encoded by seven *ChTps1* genes. The activity of ChTPS1a was highest, whereas the others varied depending on the amino acid substitution sites (Fig. 4 and Table 1). Here, we discuss the reasons for reducing or changing the enzymatic activities referring to the amino acid alignment data of ChTPS1 (Table 1), as well as the plausible 3D structural model of ChTPS1 (Supplementary Table S2 and Supplementary Fig. S4). Substitutions in ChTPS1b induced a severe (90%) decrease in the enzymatic activity. ChTPS1b has 5 substitutions and E78Q and L321I were observed only in ChTPS1b. Although E78 was hydrogen-bonded to H122 and E121, E78Q, the substitution between similar amino acids, does not seem to induce serious effects (Supplementary Table S2). L321I was observed in the molecular surface, which does not seem to affect the enzyme activity, either (Supplementary Table S2). Three other substitutions, V145M, G185E, and D317N, were also observed in ChTPS1c, e, and f. V145 exists in the internal part of the molecule and G185 locates in the loop between helices (Supplementary Table S2). These two substitutions can induce conformational changes that affect the enzyme activity. Actually, ChTPS1c, e, and f contained either V145M or G185E and the enzyme activity of these clones was lower than ChTPS1a. ChTPS1b has both substitutions of V145M and G185E, which might induced sever loss of enzyme activity. It was interesting to compare the amino acid sequences of ChTPS1c and f. The latter had K541N substitution and this mutation increased the amount of hedycaryol (Peak 1) about four times without changing that of other products.

We have prepared the R477H substitution clone, which has the same amino acid sequence of CsiTPS8, to compare the enzyme activity with ChTPS1 (ChTPS1a). R477 in a loop formed an ionic bond with E487 in a helix of ChTPS1 (Supplementary Table S2 and Supplementary Fig. S4). Hence, the R477H substitution possibly changes an interaction between the loop and the helix, which induces conformational changes that affect the enzyme activity. Actually, the product amounts of the R477H clone averaged 40 percent increase in comparison with that of ChTPS1, although ratios between products were almost same (Supplementary Fig. S6). These results, such as K541N and R477H, suggest that the amino acid substitution in the loop structure, but not in the active center, would be a useful method to modify the enzyme activity. E487 in the α-helices (483–493) forms an ionic bond with R477. And the α-helices is forming the hydrophobic interaction with the preceding α-helices (440–461) containing one of the TPS-conserved motifs (Supplemental Fig. S2). Therefore, the mutation of R477 might influence the conformation and /or position of the TPS-conserved motif. We’d like to examine the substitution effects in other TPSs, such as ZoTPS1, ZzZSS1 and ZzZSS2 (Supplemental Fig. S2). In these TPSs, amino acids corresponding with acidic E487 in ChTPS1 were lysines, whereas neighboring ones were glutamates (Supplemental Fig. S2) and amino acids corresponding with R477 were polar tyrosine or basic histidine (Supplemental Fig. S2). This would suggest that a hydrogen bond or an ionic bond between amino acids corresponding with E487 and R477 in ChTPS1 also function in these TPSs. Furthermore, basic lysines corresponding with K541 in ChTPS1 were conserved. It may be interesting to introduce substitutions into these TPSs’ amino acids corresponding with R477, E487 and K541 (e.g. E487K, K541N) and to examine whether such substitutions affect the synthesis of sesquiterpenes different from that of ChTPS1.

*ChTps1* expression analysis showed *ChTps1* was expressed primarily in flowers (Supplementary Fig. S3). This result suggested that the floral volatiles biosynthesized by ChTPS1 activity could invite pollinators to the flowers. However, different functions of floral compounds were ascribed, including defense and protection against herbivores and pathogens^1^. Because pollen and ovules are indispensable for the production of the next generation, these nutritious floral tissues may require relatively more protection from enemies^2^. Li *et al*. reported valerianol and other sesquiterpenes extracted from Chinese agarwood exhibited antibacterial activities against *Staphylococcus aureus* and *Ralstonia solanacearum*^52^. It is necessary to examine further whether valerianol and hedycaryol have such protective effects in Camellia plants.

Previous studies showed various volatile compounds in teas^29,41^; however, valerianol has not been referred to. Although there are several reports on valerianol, such as exhibiting the antibacterial activity^52^ mentioned above and the bioconversion of valerianol by the fungus *Mucor plumbeus*^53^, it has not been studied intensely on the whole. Moreover, the MS of valerianol is similar to that of guaiol; hence valerianol may always be aliased as guaiol by GC-MS analysis (Supplementary Table S1 and Fig. 2).

In conclusion, we analyzed the floral scent of *C*. *hiemalis*, and identified the *ChTps1* gene as the valerianol synthase gene, which was isolated from the *C*. *hiemalis* flower. Furthermore, we found that valerianol is included in green tea and black tea that are produced from the *C*. *sinensis* leaf buds, which are consumed widely and routinely. As far as we know, this is the first identification of a gene that can synthesize valerianol, and the first report on valerianol detection in tea. This sesquiterpenic alcohol was first isolated from valerian oil (*Valeriana officinalis*) in 1967^54^. For the 50 years since then, few studies on valerianol have been carried out. With this opportunity, that after half a century such an old study met with new findings (and pathway engineering technology) that were revealed in the present study, we expect that a revival will occur to study valerianol, e.g., through analysis of its physiological functions.

## Methods

### Plant materials

Theaceae *Camellia hiemalis*, planted in a site of Ishikawa Prefectural University, was used. Branches with several leaves and flowers were separated, frozen in liquid nitrogen, and stored at −80 °C until use.

Japanese green tea “Okumidori” produced in Kagoshima Prefecture, Japanese black tea “Benihikari” in Shizuoka Prefecture, and Indian black tea “Darjeeling” were used for the analysis of volatile compounds.

Gas chromatography (GC)-mass spectrometry (MS) analysis of floral scent and tea volatile compounds. GC-MS analysis was performed as described previously^30^. A 7890A gas chromatograph system coupled to a 5975C mass spectrometer detector (Agilent Technologies, CA, USA) was used for floral scent and tea volatiles analyses. Sample constituents were separated using a DB-WAX column (60 m, 0.25 mm ID, 0.25 μm Df). Volatile constituents were extracted using a solid phase micro-extraction (SPME) fiber (50/30 μm divinylbenzene/carboxene/polydimethylsiloxane) at 80 °C for 40 min (10 min preheating, 30 min extraction). Then, the SPME fiber was transferred and injected at 230 °C. The GC oven was programmed to start at 40 °C, hold for 10 min, then ramp at 5 °C/min to 230 °C, and hold for 12 min. Tea leaf volatiles were extracted by Stir Bar Sorptive Extraction. At first, tea leaves (3 g each) were soaked in 300 ml water and boiled down to 30 ml. 10 ml supernatant out of 30 ml was transferred to a glass vial, added 3 g NaCl, and stirred 1 hr with the polydimethylsiloxane coated stir bar at the room temperature. After the stirring, the bar was rinsed, wiped, and subjected to injection for GC-MS analysis.

### Isolation of terpene synthase cDNAs from *C. hiemalis*

Total RNA extraction, degenerate PCR and isolation of *Tps* cDNA fragments from the flowers of *C*. *hiemalis* were performed as described previously^30^.

### Cloning of cDNAs for full-length terpene synthase genes

The 3′ end cloning of a *C*. *hiemalis Tps* cDNA was performed as described previously^30^. The entire *Tps* open reading frame (ORF) was then amplified by PCR using PrimeStar GXL polymerase (Takara Bio) with primers (5′-AGACAGAAGATCTCATGGCTTCATCTCAAGTTGGTGA-3′ and 5′-GAGGTACCTCGAGTCACATGGGAATTGGATCTTCGA-3′). These primers included *Bgl*II and *Xho*I restriction endonuclease recognition sites (underlined) upstream of the initial codon and downstream of the terminal codon of the gene, respectively. PCR conditions were as follows: 94 °C for 2 min, 5 cycles of three steps (94 °C for 10 s, 55 °C for 15 s, and 72 °C for 2 min), and 20 cycles of three steps (94 °C for 10 s, 60 °C for 15 s, and 72 °C for 2 min).

The PCR products were cloned into a pGEM-T Easy vector, and their sequences were confirmed. Other molecular experiments were carried out in accordance with Sambrook & Russell^55^.

### Phylogenetic analysis

Sequence similarity of the *Tps* genes was analyzed as described previously^30,32^. Multiple alignment of plant TPSs was carried out using BioEdit free software^56^. Phylogenetic analysis of these TPSs was carried out using DNASIS Pro (Hitachi Solutions, Ltd. Tokyo, Japan). Deduced amino acid sequences of known TPSs were obtained from the National Center for Biotechnology Information (NCBI) website^57^. Known plant sesquiterpene synthases and their accession numbers were as follows: grape (*V*. *vinifera*) (*E*)-α-bergamotene synthase (VvABS), accession no. NP_001267972; (*E*)-β-caryophyllene synthase (VvBCaS), ADR74193; β-curcumene synthase (VvBCuS), ADR74200; (−)-germacrene D synthase (VvGDS), Q6Q3H3; kiwi fruit (*Actinidia deliciosa*) germacrene D synthase (AdGDS), AAX16121; shampoo ginger (*Z*. *zerumbet*) α-humulene synthase (ZzZSS1), BAG12020; β-eudesmol synthase (ZzZSS2), BAG12021; ginger (*Z*. *officinale*) germacrene D synthase (ZoGDS), AAX40665; (*S*)-β-bisabolene synthase (ZoTPS1), BAI67934; maize (*Zea mays*) terpene synthases (ZmTPS4, AAS88571; ZmTPS5, AAS88574; ZmTPS6, AAS88576; ZmTPS10, AAX99146; ZmTPS11, ACF58240); lemon basil (*Ocimum basilicum*) α-zingiberene synthase (ObZIS), AAV63788; grand fir (*Abies grandis*) δ-selinene synthase (AgAG4), AAC05727; γ-humulene synthase (AgAG5), AAC05728; western balsam poplar (*P*. *trichocarpa*) terpene synthases (PtTPS5, KF776503; PtTPS7, KF776505; PtTPS8, KF776506; PtTPS9, KF776507; PtTPS11, KF776509; PtTPS14, KF776512); sandalwood (*Santalum album*) sesquiterpene synthase (SaSesquiTPS), ACF24768; (*S*. *austrocaledonicum*) sesquiterpene synthase (SauSesquiTPS), HQ343281; (*S*. *murrayanum*) sesquiterpene synthase (SmSesquiTPS), JF746810; (*S*. *spicatum*) sesquiterpene synthase (SspiSesquiTPS), HQ343282; tobacco (*Nicotiana benthamian*) sesquiterpene synthase (NbTPS1), AHM10157; upland cotton (*Gossypium hirsutum*) sesquiterpene synthase (GhTPS1), AFQ23183; tomato (*Solanum habrochaites*) sesquiterpene synthase (ShTPS9, AEM23825; ShTPS12, AEM23826; ShTPS14a, AEM23827; ShTPS14b, AEM23829; ShTPS15b, AEM23830); (*S*. *lycopersicum*) sesquiterpene synthase (SlTPS16, AEM23831; SlTPS17, AEM23832; SlTPS31, AEM23833); camellia (*Camellia sinensis*) terpene synthase (CsiTPS4, ANB66343; CsiTPS8, ANB66333); (*C*. *brevistyla*) hedycaryol synthase (CbTPS1), BAU68096.

### GC-MS analysis of terpene products with mevalonate-pathway-engineered *E. coli*

Terpene product analysis was performed as described previously^30^. The entire *Tps* ORFs from *C*. *hiemalis* was inserted into the *Bgl*II-*Xho*I of the *E*. *coli* expression vector pRSFDuet-1 [Merck (formerly Novagen), Darmstadt, Germany], yielding the desired plasmids, named pRSF-ChTps1. The plasmid was introduced into *E*. *coli* BL21-CodonPlus (DE3) cells (Stratagene, La Jolla, CA, USA) together with plasmid pAC-Mev/Scidi/Aacl^36^, which contains the MVA-pathway gene cluster from *Streptomyces* sp. strain CL190, the *Saccharomyces cerevisiae* IPP isomerase gene (*Scidi*), and the acetoacetate-CoA ligase gene (*Aacl*) for the utilization of Li acetoacetate (LAA) as the substrate. Recombinant *E*. *coli* cells were cultured in TB medium^55^ containing kanamycin (Km; 50 μg/ml) and chloramphenicol (Cm; 30 μg/ml) at 18 °C with the addition of 0.1 mM isopropyl β-D-thiogalactopyranoside (IPTG) and 1 mg/ml LAA (Tokyo Kasei, Tokyo, Japan). The culture medium was centrifuged briefly, and the *E*. *coli* cell pellet was extracted with ethyl acetate. A terpene product in the extract was concentrated about fivefold by decompression. GC-MS analysis was performed on Shimadzu GCMS-QP2010 (Shimadzu, Kyoto, Japan) equipped with a DB-5ms capillary column (0.25 mm internal diameter ×0.25 μm ×30 m, Agilent Technologies)^36^. Split injections (1 μL) were made at a ratio of 10:1 with an injection room temperature of 260 °C. The heating-up program of the column oven was 40 °C (held for 1 min), 4 °C min^−1^ increments up to 120 °C, and 15 °C min^−1^ increments up to 260 °C (held for 4 min). Mass spectra were monitored in the mass range of m/z 45–250 with an electron voltage at 1.15 kV and an interface temperature at 280 °C.

For comparing the product amounts of the 7 *ChTps1* clones, 1 ml dodecane, which would trap terpene products, was added to 5 ml TB medium before the inoculation and 1 μL dodecane was analyzed by GC-MS when culturing was finished. The heating-up program of the column oven was 130 °C (held for 10 min), 2.5 °C min^−1^ increments up to 170 °C, and 15 °C min^−1^ increments up to 260 °C (held for 4 min).

### Cultivation of recombinant *E. coli*, purification and spectroscopic analyses of a *ChTPS1* product

The *E*. *coli* cells transformed with plasmids pRSF-ChTps1 and pAC-Mev/Scidi/Aacl were grown in LB medium^55^ containing Km and Cm at 25 °C with reciprocal shaking for 7–8 h until the absorbance at OD 600 nm had reached approximately 1.0. One milliliter of this culture was inoculated into 100 ml of TB medium in a Sakaguchi flask containing 50 μg/ml Km, 30 μg/ml Cm, and 1 mg/ml LAA, and cultured at 37 °C with rotary shaking (180 rpm) for 3–4 h until the absorbance at OD 600 nm reached approximately 1.0. After cooling the flask to 20 °C, 25 μM IPTG (final concentration) and 20 ml *n*-octane were added to each flask, and cultured at 20 °C with rotary shaking (180 rpm) for 2 days.

The contents of 10 flasks [1 L culture and *n*-octane layer (200 ml)] were added to *n*-hexane (200 ml), and partitioned between alkane (*n*-octane + *n*-hexane) and H_2_O. The alkane layer was washed with 400 ml of 50% alkaline MeOH [MeOH (150 ml) +0.1 N KOH (150 ml)] twice, and concentrated to a small volume (1.0 ml). The concentrated alkane was subjected to silica gel column chromatography (silica gel 60, 20 mm × 200 mm) filled with *n*-pentane and developed with *n*-pentane (300 ml) → *n*-pentane-EtOAc (5:1) (300 ml) stepwisely. The eluate solution was fractionated for each 7 ml (fr. 1–30 (*n*-pentane fractions) and fr. 31–60 (*n*-pentane-EtOAc (5:1) fractions) in this silica gel chromatography, and fr. 39–44 which contain Rf 0.30 silica gel TLC spot (solvent: *n*-pentane-EtOAc (5:1)) was combined and concentrated to dryness to afford a pure terpene product (valerianol 10.2 mg).

### NMR spectroscopic data of valerianol

NMR spectra were measured by an AVANCE400 (Bruker BioSpin, Karlsruhe, Germany) in CDCl_3_, using the residual solvent peak as an internal standard (δ_C_ 77.0, δ_H_ 7.26 ppm).

^1^H NMR (CDCl_3_) δ: 0.90 (d *J* = 6.8 Hz, 3H, H-11), 0.95 (m, 1H, H-8b), 1.11 (s, 3H, H-12), 1.16 (s, 6H, H-14 and H-15), 1.15 (m, 1H, H-6b), 1.36 (m, 1H, H-3b), 1.40 (m, 1H, H-4a), 1.56 (m, 1H, H-4), 1.68 (m, 1H, H-3a), 1.70 (m, 1H, H-7), 1.82 (m, 1H, H-2b), 1.88 (m, 1H, H-8a), 1.90 (m, 1H, H-2a), 2.04 (m, 1H, H-9b), 2.26 (m, 1H, H-9a), 5.33 (m, 1H, H-1). ^13^C NMR (CDCl_3_) δ: 15.5 (C-11), 23.7 (C-2), 25.3 (C-12), 26.9 (C-3), 27.0 (C-14), 27.1 (C-15), 29.6 (C-8), 32.5 (C-9), 35.2 (C-6), 37.7 (C-5), 39.3 (C-4), 44.2 (C-7), 119.0 (C-1), 143.1 (C-10).

### Expression analysis of *ChTps1*

*ChTps1* gene expression was analyzed by the RT reaction and following PCR. Three replicates of total RNAs were extracted from the flower, leaf, and stem tissues of *C*. *hiemalis* and were reverse-transcribed with oligo-dT primer using PrimeScript RT Master Mix (Takara Bio). The PCR amplification was performed using Ex Taq HS (Takara Bio). PCR conditions were as follows: 95 °C for 2 min, 35 cycles of three steps (95 °C for 10 s, 66 °C for 20 s, and 72 °C for 15 sec). The following primers were used for *ChTps1*detection: 5′-CTACAGGGCTTGACTGCTAC-3′ and 5′-CAGTTGGCTTAAGCATTTCT-3′, and for *C*. *hiemalis* actin-like gene amplification: 5′-GCAAGAGCTGGAGACTGCG-3′ and 5′-GTGCTTAGGGATGCAAGGATAGAT-3′.

Images of gel electrophoresis were cropped by Adobe Photoshop Elements 8.0.

### Inhibitory activity assay of sesquiterpenes on induction of EBV-EA activation by TPA

This assay was carried out by the method described^42^.

### Accession numbers of *ChTps1*

The nucleotide sequences reported in this paper were submitted to DDBJ under the accession numbers LC212976 (*ChTps1a*), LC212977 (*ChTps1b*), LC212978 (*ChTps1c*), LC212979 (*ChTps1d*), LC212980 (*ChTps1e*), LC212981 (*ChTps1f*), and LC212982 (*ChTps1g*).

### Ethical Statement

This article does not contain any studies with human participants or animals performed by any of the authors.

## Electronic supplementary material

Supplementary Information

## Data Availability

The data supporting the findings of this study are available upon reasonable request to the corresponding author.
