# Supplementary material for: Identification of novel sesquiterpene synthase genes that mediate the biosynthesis of valerianol, which was an unknown ingredient of tea
Source: Sci Rep. 2018 Aug 20;8:12474. doi: 10.1038/s41598-018-30653-w (PMC6102311; doi:10.1038/s41598-018-30653-w)
Supplement: Supplementary file 1 — Supplementary Information [file 41598_2018_30653_MOESM1_ESM.pdf]

Supplementary Information

Identification of novel sesquiterpene synthase genes that mediate the biosynthesis of valerianol, which was an unknown ingredient of tea

Jun-ichiro Hattan[1], Kazutoshi Shindo[2], Tetsuya Sasaki[3], Fumina Ohno[1], Harukuni Tokuda[4], Kazuhiko Ishikawa[5], and Norihiko Misawa[1*]

[1] Research Institute for Bioresources and Biotechnology, Ishikawa Prefectural University, 1-308 Suematsu, Nonoichi-shi, Ishikawa 921-8836, Japan
[2] Department of Food and Nutrition, Japan Women's University, 2-8-1 Mejirodai, Bunkyo-ku, Tokyo 112-8681, Japan
[3] Industrial Research Institute of Ishikawa, 2-1 Kuratsuki, Kanazawa-shi, Ishikawa 920-8203, Japan
[4] Department of Complementary and Alternative Medicine, Clinical R&D, Graduate School of Medical Science, Kanazawa University, 13-1, Takara-machi, Kanazawa-shi, Ishikawa 920-8640, Japan
[5] National Institute of Advanced Industrial Science and Technology, 1-8-31 Midorigaoka, Ikeda-shi, Osaka 563-8577, Japan

[*]To whom correspondence should be addressed. E-mail: n-misawa@ishikawa-pu.ac.jp
Phone: 81 76 227 7525, Fax: 81 76 227 7557 (Ishikawa Prefectural University)

Supplementary Table S1 Terpenes and their ratio in the volatile compounds trapped by solid phase micro-extraction from the flowers of *Camellia hiemalis*

Component	content (%)
cis-Linalool oxide	1.37
Linalool	5.15
β-Elemene	0.08
α-Terpineol	0.11
Epoxylinalool	0.79
Elemol	0.1
Hexahydrofarnesyl acetone	0.89
Eugenol	80.94
Guaiol	0.39
α-Muurolene	0.1
α-Eudesmol	0.07
β-Eudesmol	0.14
Total	90.13

Supplementary Table S2 Classification of amino acid substitutions observed in ChTPS1 clones

amino acid	location and interaction in the molecule	expected effects of substitutions on the enzyme activity
W42	surface	no effect
A68	surface, entrance of the cleft	unclear
E78	hydrogen bonding with H122, E121	E78Q has no effect
G139	loop*	G139C has no effect
V145	internal	unclear
G185	loop*	possibly changes the conformation
S290	surface	no effect
V295	internal	unclear
D317	surface	no effect
L319	internal	unclear
L321	surface	no effect
D365	surface	no effect
A395	surface	no effect
E399	surface	no effect
A443	internal	unclear
R477	surface, loop*, forming an ionic bond with E487	R477H possibly change the loop structure
M503	internal	unclear
K541	surface, loop*, hydrogen bonding with D542	K541N possibly change the peak1 productivity

*loop means β-turn

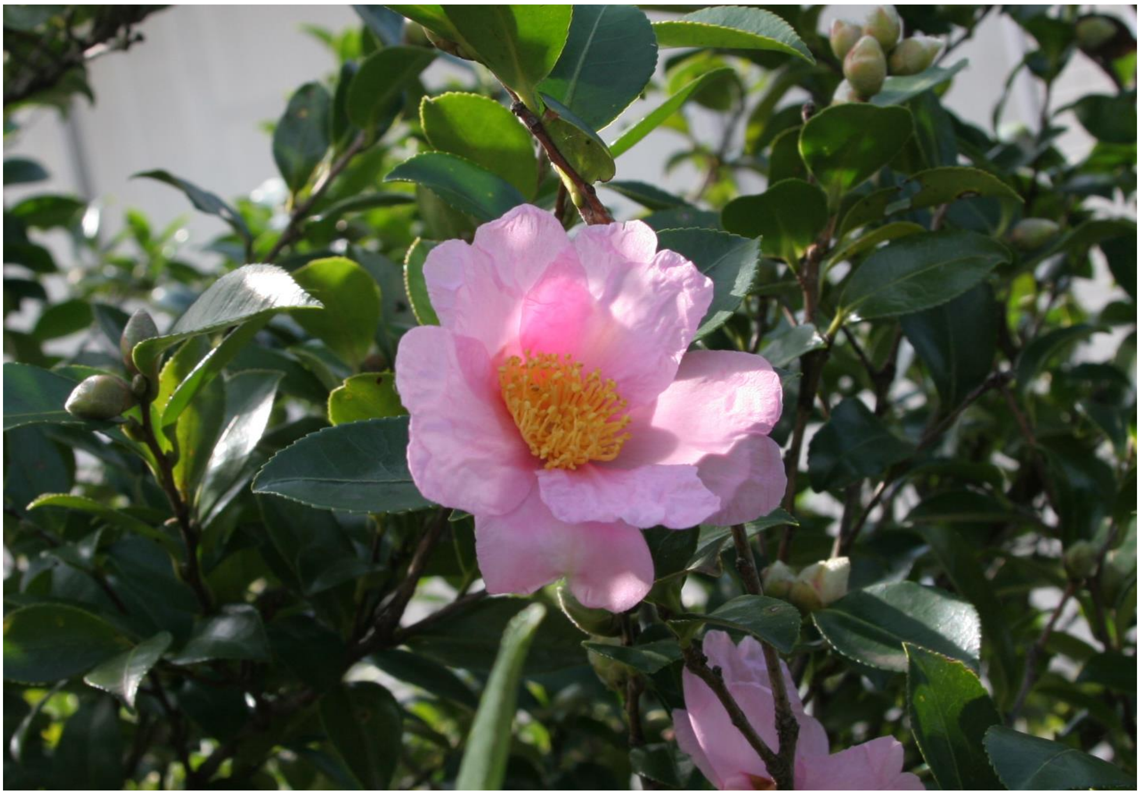

Supplementary Fig. S1 Flowers of scented Camellia, *Camellia hiemalis*

```
ZoTPS1  MELVDTPSLEVFEDVVVDRQVAGFDPSFWGDYFITN----QKSQSEAWMNERAEELKNEVRSMFQNV-TG 65
ZzZSS1  MERQ-SMALVGDKEEII-RKSFEYHPTVWGDYFIRNYSCLPLEK--ECMIKRVEELKDRVRNLFEET-HD 65
ZzZSS2  MEKQ-SLTFDGDEEAKIDRKSSKYHPSIWGDYFIQNSSLTHAKESTQRMIKRVEELKVQVKSMFKDT-SD 68
CbTPS1  MASS-QVGDMVNGNAEPTRHLAKFPPSLWGDRFT---SFTLDKQLWDKYGNEIEVLKEQVRSMVVAGGRK 66
ChTPS1  MASS-QVGDMVNGNAEPTRHLAKFPPSLWGDRFT---SFTLDKQLWDKYGNEIEVLKEQVRSMVVAGGRK 66

ZoTPS1  ILQTMNLIDTIQLLGLDYHFMEEIAKALDHLKD--VDMSKYGLYEVALHFRLLRQKGFNISSDVFKKYKD 133
ZzZSS1  VLQIMILVDSIQLLGLDYHFEKEITAALRLIYE--ADVENYGLYEVSLRFRLLRQHGYNLSPDVFNKFKD 133
ZzZSS2  LLQLMNLINSIQMLGLDYHFENEIDEALRLIYE--VDDKSYGLYETSLRFQLLRQHGYHVSADIFNKFKD 136
CbTPS1  AAEQINLINVLERLGVSYHFEKEIEEQLEQLFAKFEDNEDYDLFTIALHFRIFRQHGYKMSCDVFNKFRD 136
ChTPS1  AAEQINLINVLERLGVSYHFEKEIEEQLEQLFAKFEDNEDYDLFTIALHFRIFRQHGYKMSCDVFNKFRD 136

ZoTPS1  KEGKFMEELKDDAKGLLSLYNAAYFGTKEETILDEAISFTKDNLTSLLK--DLNPPFAKLVSLTLKTPIQ 201
ZzZSS1  DKGRFLPTLNGDAKGLLNLYNAAYLGTHEETILDEAISFTKCQLESLLG--ELEQPLAIEVSLFLETPLY 201
ZzZSS2  DNGSFISSLNGDAKGLLSLYNVSYLGTHGETILDEAKSFTKPQLVSLMS--ELEQSLAAQVSLFLELPLC 204
CbTPS1  SNGEFKETVSNDVRGMLSLYEATYLKIRGEGFLDEAHAFTIAQLESLVGGPHLSSDLSEQVMHALKQSIH 206
ChTPS1  SNGEFKETVSNDVQGMLSLYEATYLKIRGEGFLDEAHAFTIAQLESLVGGPHLSSDLSEQVMHALKQSIH 206
                     +
                                                                        ***
ZoTPS1  RSMKRIFTRSYISIYQDEPTLNETILELAKLDFNMLQCLHQKELKKICAWWNNLNLDIMHLNFIRDRVVE 271
ZzZSS1  RRTRRLLVRKYIPIYQEKVMRNDTILELAKLDFNLLQSLHQEEVKKITIWWNDLALTKS-LKFARDRVVE 270
ZzZSS2  RRNKILLARKYILIYQEDAMRNNVILELAKLNFNLLQSLYQEELKKISIWWNDLAFAKS-LSFTRDRVVE 273
CbTPS1  RGFPRLEAKHFISFYEKDAARNETLLRLAKLDFNQLQLSHREELCHIFRWWKELDLISK-VPYARDRAVE 275
ChTPS1  RGFPRLEAKHFISFYEKDASRNETLLRLAKLDFNQLQLSHREELCHIFRWWKELDLISK-VPYARDRAVE 275
                           +
                                       *****
ZoTPS1  CYCWSMVIRHEPSCSRARLISTKLLMLITVLDDTYDSYSTLEESRLLTDAIQRWNPNEVDQLPEYLRDFF 341
ZzZSS1  CYYWIVAVYFEPQYSRARVITSKAISLMSIMDDIYDNYSTLEESRLLTEAIERWEPQAVDCVPEYLKDFY 340
ZzZSS2  GYYWVLTIYFEPQHSRARVICSKVFAFLSIMDDIYDNYGILEECTLLTEAIKRWNPQAIDGLPEYLKDYY 343
CbTPS1  CFFWSTCAYYEPQHSVGRAVLTKIVLLLSVTDDTYDAYGTYDELKLYTNAVQRWDASAMDELPDYMKTLY 345
ChTPS1  CFFWSTCAYYEPQHSVGRAVLTKIMLLLSVTDDTYDAYGTYDELKLYTNAVQRWDVSAMDELPDYMKALY 345
                                +                              +           +

ZoTPS1  LKMLNIFQEFENELA-PEEKFRILYLKEEWKIQSQSYFKECQWRDDNYVPKLEEHMRLSIISVGFVLFYC 410
ZzZSS1  LKLLKTYKDFEDELE-PNEKYRIPYLQEEIKVLSRAYFQEAKWGVERYVPALEEHLLVSLITAGYFAVAC 409
ZzZSS2  LKLLKTFEEFEDELE-LNEKYRMLYLQDEVKALAISYLQEAKWGIERHVPSLDEHLHNSLISSGSSTVIC 412
CbTPS1  RALLNVYDEVERDLAKQGRAYGVHHSKEAFKEIVRSYEIEAEWFKEGYVASFEEYMKNALVTSTGRLHTT 415
ChTPS1  RALLNVYDEVERDLAKQGRDYGVHHSKEAFKEIVRSYEIEAEWFKEGYVASFEEYMKNALVTSTGRLHTT 415
                           +
                                            *********
ZoTPS1  GFLSGMEEAVATKDAFEWFASFPKIIEACATIIRITNDITSMEREQKRAHVASTVDCYMKEYGTSKDVAC 480
ZzZSS1  ASYVGLG-EDATKETFEWVASSPKILKSCSIHCRLMDDITSHQREQERDHFASTVESYMKEHGTSAKVAC 478
ZzZSS2  ASFVGMG-EVATKEVFDWLSSFPKVVEACCVIGRLLNDIRSHELEQGRDHTASTVESYMKEHDTNVDVAC 481
CbTPS1  SCFMGLEADVATTEAFEWILTKPKMVAASGAIGRLVDDVMSNDEEQERGHVATGLDCYMKQHGVSKQEAI 485
ChTPS1  SCFMGLEADVATTEAFEWILTKPKMVAASGAIGRLVDDVMSHDEEQERGHVATGLDCYMKQRGVSKQEAI 485
                                                 +                   +

ZoTPS1  EKLLGFVEDAWKTINEELLTETGLSREVIELSFHSAQTTEFVYKHVDAFTEPNTTMKENIFSSLLVHPIPI 550
ZzZSS1  EKLQVMVEQKWKDLNEECLRPTQVARPLIEIILNLSRAMEDIYKHKDTYTNSNTRMKDNVSLIFVESFLI 548
ZzZSS2  EKLREIVEKAWKDLNNESLNPTKVPRLMIERIVNLSKSNEEIYKYNDTYTNSDTTMKDNISLVLVESCDY 551
CbTPS1  VELYKMIENAWRDINEEMLKPTAISMKLLIHVLNLSRISDVVYKYVDGYTHP-EIIKDHVISLFEDPIPM 554
ChTPS1  VELYKMIENAWRDINEEMLKPTAISMKLLIRVLNLSRISDVVYKYVDGYTHP-EIIKDHVISLFEDPIPM 554
                                      +

ZoTPS1  --- 550
ZzZSS1  --- 548
ZzZSS2  FNK 554
CbTPS1  --- 554
ChTPS1  --- 554
```

Supplementary Fig. S2 Amino acid alignment of sesquiterpene synthases derived from *Zingiber* plants and *Camellia* plants

The deduced amino acid sequences of the *Zingiber* terpene synthases (TPSs) together with *Camellia* TPSs, isolated by our group, were aligned. The TPS-conserved motifs RDR, DDxxD, and NSE/DTE [(N/D)Dxx(S/T)xxxE] are indicated by asterisks. Amino acids conserved in all TPSs are shown in black boxes, whereas amino acids that are not conserved between CbTPS1 and ChTPS1

are indicated by +. Arrows indicate the sequences corresponding to the degenerate primers. Three amino acids encircled by a simple square would correspond to E487 in ChTPS1. ZoTPS1, *Zingiber officinale* β-bisabolene synthase[32]; ZzZSS1, *Z. zerumbet* α-humulene synthase[34]; ZzZSS2, *Z. zerumbet* β-eudesmol synthase[33]; CbTPS1, *Camellia brevistyla* hedycaryol synthase[30].

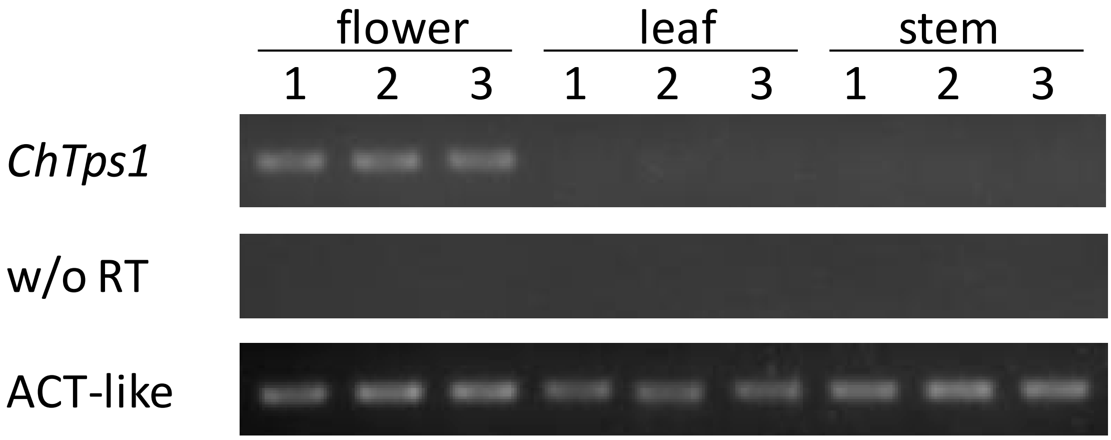

Supplementary Fig. S3 Expression analysis of the *Camellia hiemalis ChTps1* gene by reverse transcription (RT)-polymerase chain reaction (PCR). Total RNAs were extracted from 3 independent samples (1-3) of each tissue (flowers, leaves, and stems) and submitted for RT-PCR. Total RNA without a treatment of reverse transcriptase was used as the template for the 'w/o RT' reaction. An actin-like (ACT-like) gene of *C. hiemalis* was amplified as an internal control. Nine samples (three replicates of each tissue) in each row (*ChTps1*, w/o RT, ACT-like) were simultaneously loaded into one gel and electrophoresed.

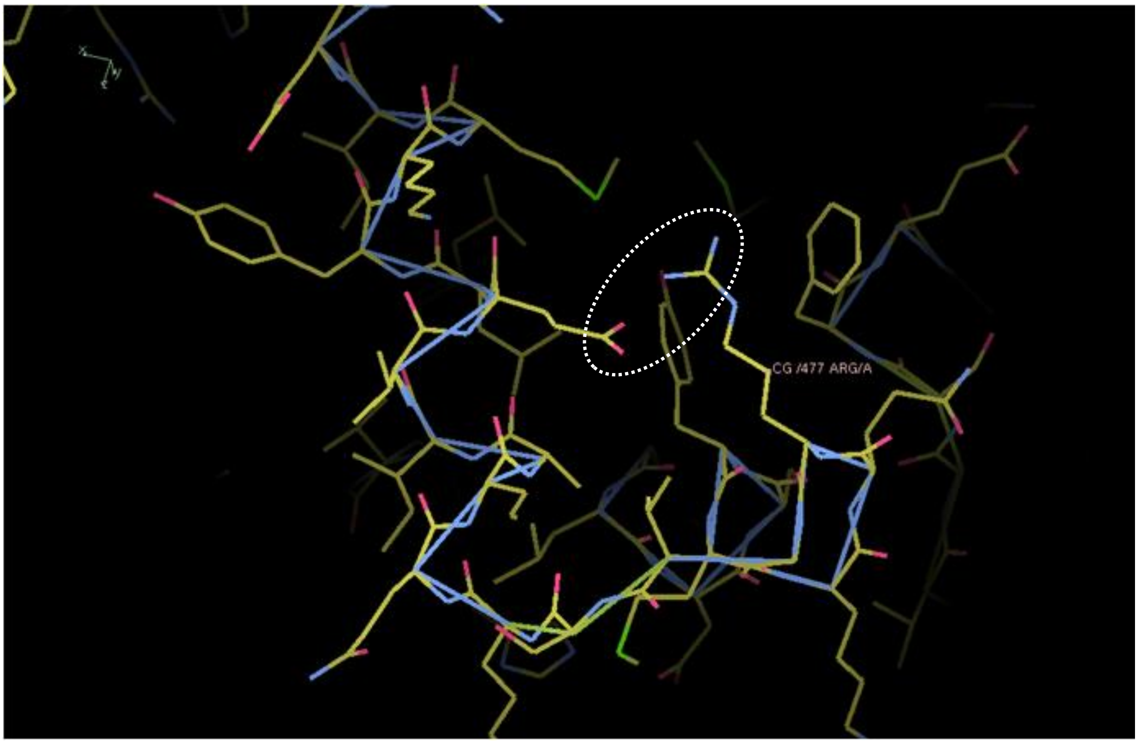

Supplementary Fig. S4 Schematic diagram of the loop structure around R477. An ionic bond between R477 and E487 was predicted in this model (dotted circle). The atoms were colored as C: yellow, N: blue, O: red, S: green. The backbone of polypeptide was shown in blue line.

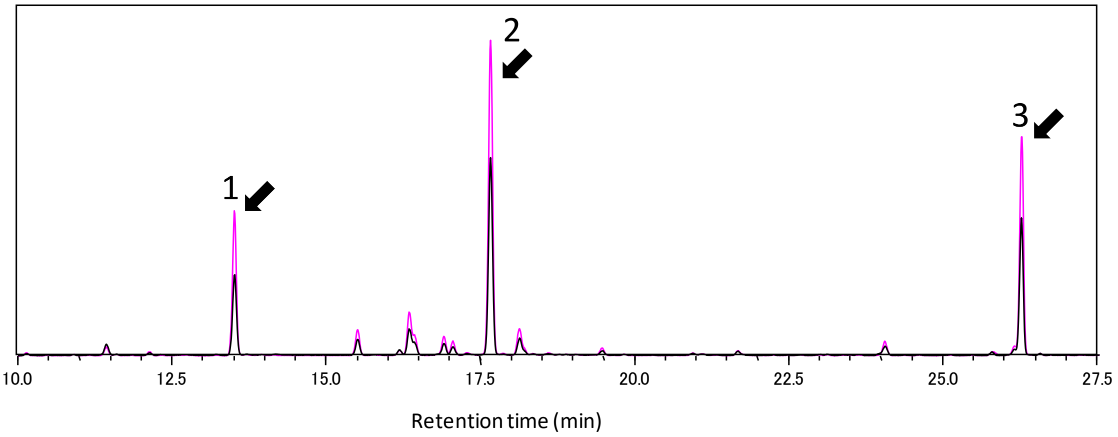

Supplementary Fig. S5 Comparison of the *Camellia hiemalis* ChTPS1 and ChTPS1 (R477H) products. Volatile compounds biosynthesized by ChTPS1 and ChTPS1 (R477H) were extracted by decane and analyzed by GC-MS. The chromatograms were overlapped in the same figure (ChTPS1: black, ChTPS1(R477H): pink). Three peaks correspond to those in Fig. 2a.

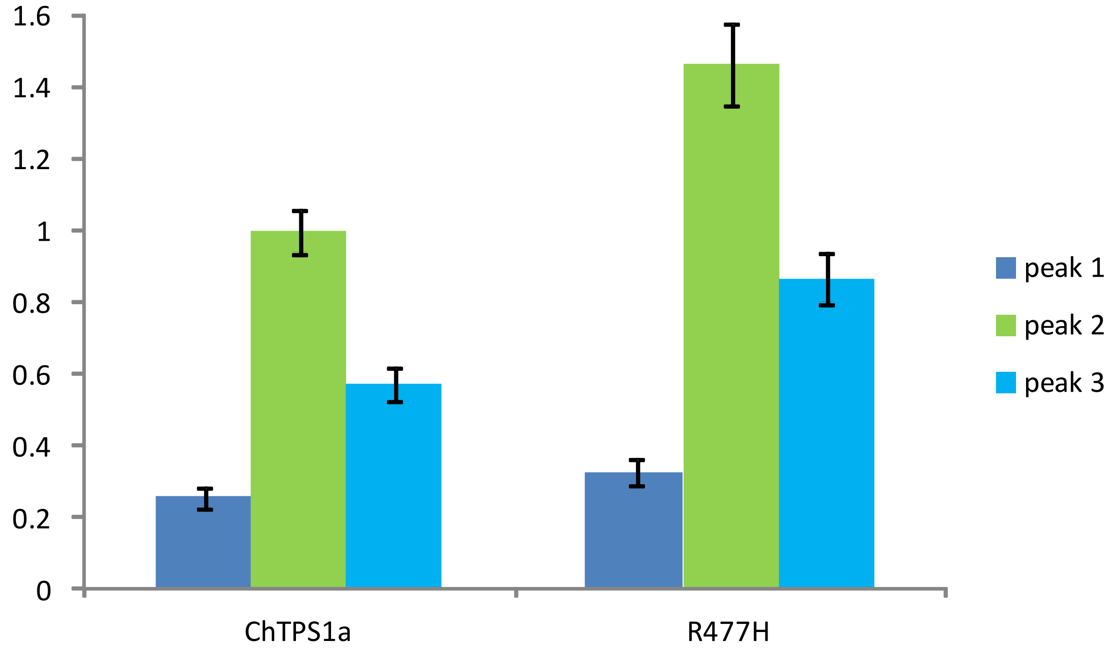

Supplementary Fig. S6 Comparison of the peak areas obtained by GC-MS analysis of *ChTps1a* and *ChTps1*(R477H) clone's products. Three peak areas, shown in Fig. 2a (Peaks 1-3), of total ion chromatogram were obtained by the analysis. Relative abundance of each peak was depicted when the Peak 2 area of *ChTps1a* clone was set to 1. Bars represent means ± standard deviation of three replicates.

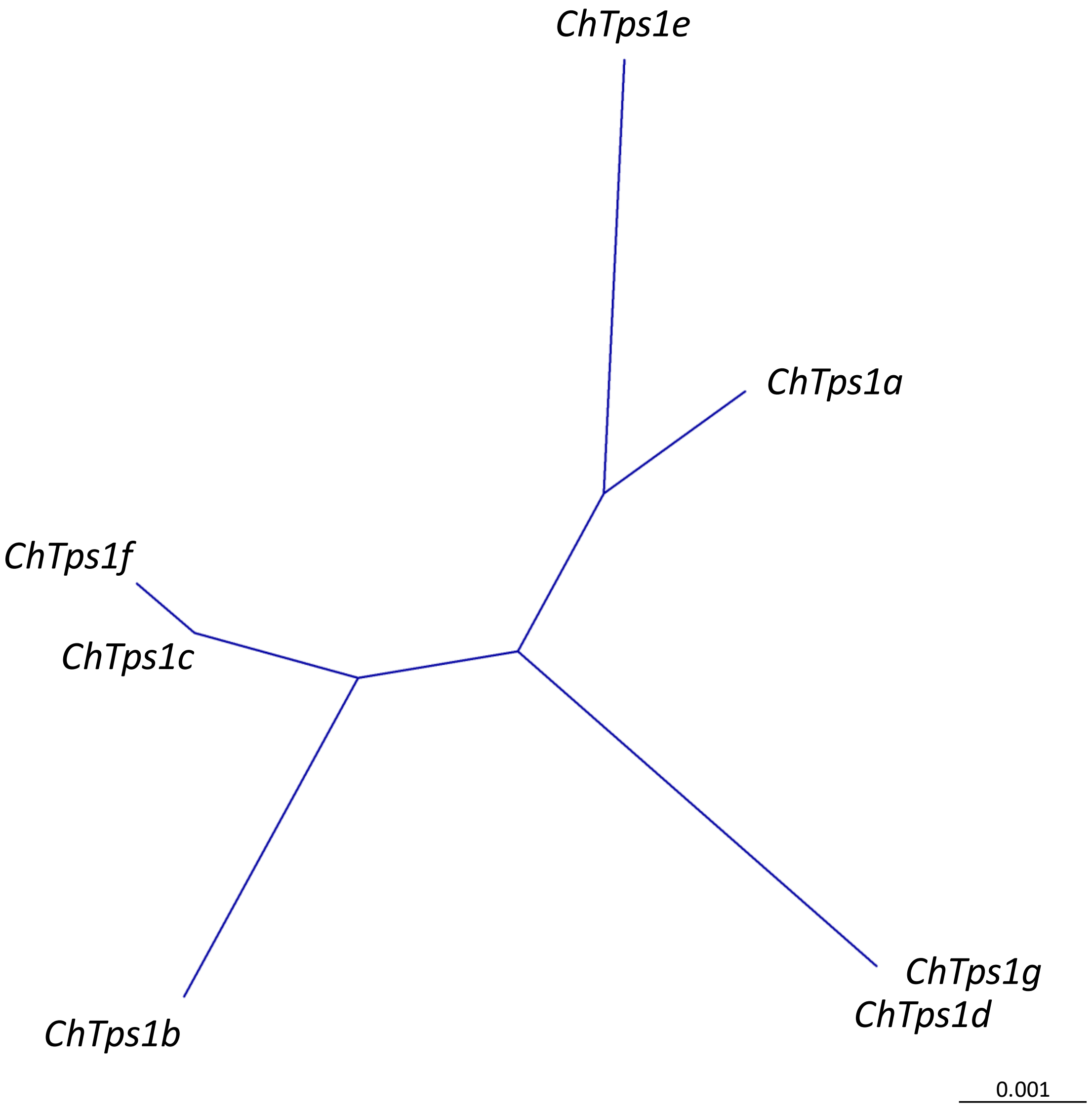

Supplementary Fig. S7 Phylogenetic positions of the *ChTps1a-g* genes. The nucleotide sequences of these seven genes were compared phylogenetically.
